# Research on Health Disparities Related to the COVID-19 Pandemic: A Bibliometric Analysis

**DOI:** 10.3390/ijerph19031220

**Published:** 2022-01-22

**Authors:** Keng Yang, Hanying Qi

**Affiliations:** 1 Institute of Economics, Tsinghua University, Beijing 100084, China; yangkengok@163.com; 2 One Belt-One Road Strategy Institute, Tsinghua University, Beijing 100084, China; 3 The New Type Key Think Tank of Zhejiang Province “Research Institute of Regulation and Public Policy”, Zhejiang University of Finance and Economics, Hangzhou 310018, China; 4 China Institute of Regulation Research, Zhejiang University of Finance and Economics, Hangzhou 310018, China

**Keywords:** health disparities, health inequalities, COVID-19, public health emergency, public health regulatory policy, bibliometric analysis

## Abstract

With the outbreak of the 2019 coronavirus (COVID-19) pandemic, the issue of increasing health disparities has received a great deal of attention from scholars and organizations. This study analyzes 2282 papers on COVID-19-related health disparities that have been retrieved from the WOS database, with 58,413 references. Using bibliometric analysis and knowledge mapping visualizations, the paper focuses on the academic structure and research trends by examining the research distribution of countries, journals and authors, keywords, highly cited articles, and reference co-citation. The results show that the United States has contributed the most, and the *International Journal of Environmental Research and Public Health* has published the largest number of papers on this topic. As for the core authors, Michael Marmot is the most productive. Issues such as racial health, mental health, and digital health disparities have been the trending topics of the COVID-19-related health disparities. The research directions include the features, factors, and interventions of health disparities under the influence of COVID-19. As such, this study provides literature support and suggestions to investigate COVID-19-related health disparities. The findings of the paper also remind public health regulators to consider factors of health disparities when developing long-term public health regulatory policies related to the pandemic.

## 1. Introduction

Health disparities research has attracted widespread attention from scholars and policy makers in public health since the 19th century [1]. There is no consensus on the definition of health disparities, but all the definitions share a common theme: health differences among groups. For example, a health disparity was defined by Healthy People (2020) as “a particular type of health difference that is closely linked with social, economic, and/or environmental disadvantage”. (U.S. Department of Health and Human Services. The Secretary’s Advisory Committee on National Health Promotion and Disease Prevention Objectives for 2020. Phase I report: Recommendations for the framework and format of Healthy People 2020 (Internet). Section IV: Advisory Committee findings and recommendations (cited 6 January 2010). Available from: http://www.healthypeople.gov/sites/default/files/PhaseI_0.pdf accessed on 15 November 2021). The Center for Disease Control and Prevention (CDC) defined health disparities as “preventable differences in the burden of disease, injury, violence, or opportunities to achieve optimal health that are experienced by socially disadvantaged populations”. (Available from: https://www.cdc.gov/healthyyouth/disparities/index.htm#1 accessed on 15 November 2021) Numerous studies have examined the social, environmental and biological factors that contribute to health disparities, such as race, ethnicity, gender, age, education, mental health, geographical area, socioeconomic status, and so on [2,3]. These studies have aimed to eliminate disparities and to achieve health equity among all groups [4].

Several articles focused on the bibliometric analysis of health disparities or health inequalities. Almeida-Filho et al. (2003) conducted a bibliometric analysis of health inequalities research in Latin American and Caribbean countries from 1971 to 2000 [5]. They identified the types of research and factors related to inequity. The factors include poverty, socioeconomic stratification, economic development, living conditions, the social relations of production, the class structure of society, and gender/ethnic affiliation [5]. Bouchard et al. (2015) analyzed 49,294 references (1966–2014) and 25 of the most-cited papers on health inequality/disparity. They identified the evolution of research themes over time as well as the author with the highest citation frequency [6]. Cash-Gibson et al. (2018) analyzed the global health inequalities in the similar time period (1966–2015). They mainly analyzed the volume and distribution of production, as well as the international collaborations and co-authors network [7]. Arul and Mesfin (2017) identified the content, journal distribution, and the common topics of the top 100 cited articles in health care disparities based on bibliometric analysis. The most common topics included the disparities in cancer, mental health, and the relationship between physicians and minority patients [2]. These articles have supported the research agenda for health disparities.

Although health disparities are not a new topic, the rapid spread of the 2019 coronavirus (COVID-19) has caused health disparities to become more acute and urgent globally. For example, in the United States, the rates of illness and death are higher in disadvantaged populations than in other populations due to COVID-19. These disparities precipitate social instability, in forms such as incidents of police brutality and increases in hate crimes against Asians. (The report is available at: https://www.kff.org/racial-equity-and-health-policy/issue-brief/disparities-in-health-and-health-care-5-key-question-and-answers/ accessed on 15 November 2021). Many studies have focused on health disparities during the COVID-19 pandemic [8,9]. They are dedicated to uncovering the impact of COVID-19 and to developing sound prevention, treatment and regulatory policies in public health in order to reduce or eliminate growing health disparities [10]. 

Few studies have analyzed the literature on health disparities during the COVID-19 pandemic. A systematic bibliometric analysis will help clarify the academic structure and the research trends of COVID-19-related health disparities. The academic structure could provide an organized understanding of a given research area. The research trends may be able to enlighten scholars to delve deeper into issues such as how COVID-19 affects health disparities, what manifestations of health disparities have emerged during the pandemic, and how the pandemic-related health disparities should be reduced on a global scale. In light of these considerations, we present a systematic bibliometric analysis of the literature.

The article makes three contributions to existing research. Firstly, this study is the first bibliometric analysis concerning COVID-19-related health disparities. Secondly, this study provides a comprehensive analysis of the academic and intellectual structure of research on health disparities related to the COVID-19 pandemic. Thirdly, the analysis of core authors, journals, papers, and research directions that is presented in this study can provide a research base and new perspectives for future studies on health disparities within the context of public health emergencies.

The article consists of three main parts. Section 2 concerns methods and data collection. Section 3 presents the results of the bibliometric analysis of scientific networks, including categories, countries, journals, authors, co-occurrences of keywords, highly cited articles, and co-citations. Section 4 contains a discussion of the results and final conclusions.

## 2. Methods and Data

### 2.1. Methods

The methods used in this paper include bibliometric analysis and the mapping of knowledge domains. The concept of bibliometrics was introduced by Pritchard (1969) [11], who defined it as “the application of mathematics and statistical methods to books and other media of communication”. The methodology is a subfield of scientometrics, used to objectively evaluate research results [12]. It helps researchers understand the intellectual structure, usefulness of papers or journals, research trends, and emergence of new research fields in a given research area by analyzing the properties of publications [12,13]. The properties usually include distribution of scientific productivity (e.g., authors, institutions, countries, and journals), citation status of publications, and scientific networks (e.g., scientific collaboration and disciplines relations) [12,14]. This method has been widely used by scholars in many scientific areas [12,15,16,17,18] and in the ranking of research organizations [19]. Publications that perform bibliometric analysis could well inform and enlighten policymakers, scientists, or other stakeholders [19]. 

The mapping of knowledge domains is a field that is a method of scientometrics [20]. It is a quantitative and visual research method for analyzing scientific activities and publications [21,22,23]. We used science mapping tools for the scientometric analysis. Science mapping can reveal knowledge regarding structures, connections, and interactions by establishing scientific networks. Science mapping tools include BibExcel, CitNetExplorer, HistCite, Leydesdorff Toolkit, SCI of SCI, Network Workbench, VOSviewer, and CiteSpace [24,25]. Among them, VOSviewer and CiteSpace are used frequently. VOSviewer was developed by van Eck and Waltman [24] at the Centre for Science and Technology Studies at Leiden University in the Netherlands. Comparisons have shown that VOSviewer can provide clear visualizations of keyword co-occurrence networks. Accordingly, we used VOSviewer (version 1.6.16) to execute the bibliometric analysis. CiteSpace was developed by Chen [26]. We used it (version 5.8.R1) to produce the dual map of citing articles and cited references. 

As for the methods of data analysis, we mainly used the following bibliometric-related analysis methods in this paper to explore academic structure. We did a series of statistical descriptions of the publications by analyzing the distribution of research categories, countries or regions, journals, and authors. At the same time, we evaluated the contributions and cooperation of countries or regions, journals, and authors by investigating citation status, co-authorship network and co-citation network. Furthermore, for the research trends, we introduced the analysis of dual-map overlay, co-occurrence of keywords, highly cited documents, and reference co-citation network. 

### 2.2. Data Collection

We collected articles from the Web of Science (WOS) database. The data were retrieved on 31 October 2021. Since 2020, 2282 papers on the relationship between COVID-19 and health disparities have been published, with 58,413 cited references and a total of 17,995 citations.

The search query method mainly concerns the screen steps in Chen’s work [20]. We combined multiple topical search queries to generate the data. The queries included keywords related to health disparities and COVID-19. Various health disparity-related terms are often used by scholars interchangeably. These include “health inequalities”, “health equalities”, “health inequity”, “health equity”, and so on. In some studies, they have similar connotations; other studies distinguish between health disparities (or inequalities) and health equity. However, the works of scholars and practitioners all aim to reduce and eliminate disparities and to achieve health equity among all groups [4]. Therefore, the keywords that we used for health disparities are “health inequality”, “health equality”, “health inequity”, “health equity”, and related synonyms. Our search strategy was as follows:

(1) We retrieved articles about COVID-19. The query command for Set #1 was TS = (“COVID19” OR “COVID-19” OR “COVID-2019” OR “coronavirus disease 2019” OR “SARS-CoV-2” OR “sars2” OR “2019-nCoV” OR “2019 novel coronavirus” OR “coronavirus disease 2019” OR “coronavirus disease-19” OR “novel coronavirus” OR “SARS-CoV-2019” OR “SARS-CoV-19” OR “COVID” OR “nCoV”).
(2) We retrieved the literature on health disparities. The topical search query covered synonyms of the term “health disparities”. The query command for Set #2 was TS = (“health* disparit*” OR “health care disparit*” OR “disparit* of health*” OR “disparit* of health care”).
(3) We searched for articles that are related to health equality or equity. The query command for Set #3 was TS = (“health* equalit*” OR “health care equalit*” OR “equalit* of health*” OR “equalit* of health care” OR “health* equit*” OR “health care equit*” OR “equit* of health*” OR “equit* of health care”).
(4) We retrieved all of the literature on health inequality or inequity. The topical search query covered synonyms of the term “inequality”. The query command for Set #4 was TS = (“health* inequalit*” OR “health care inequalit*” OR “inequalit* of health*” OR “inequalit* of health care” OR “health* inequit*” OR “health care inequit*” OR “inequit* of health*” OR “inequit* of health care”).
(5) We combined the foregoing sets through the command (((#4) OR #3) OR #2) AND #1). This command yielded 2282 bibliographic records that were obtained from the database.

## 3. Results of the Bibliometric Analysis

### 3.1. Analysis of the Research Categories

In total, the 2282 articles were published in 146 research areas according to the WOS. Table 1 displays the top 10 research categories that are related to health disparities and COVID-19. Health care sciences and services is the research category that contained the highest number of publications (1620, 70.99%). This field is a cross-cutting discipline that usually focuses on issues in public health, environmental and occupational health, medical informatics, and business economics [27]. The COVID-19 pandemic has significantly impacted public health governance systems and health care delivery systems worldwide. Health disparities have not only increased but also begun to exhibit new features while posing new problems in different fields. This process is evident in several other research directions. For example, the research directions of public environmental occupational health (1573, 68.93%) and infectious disease (1481, 64.899%) are the research areas that contained the second and third highest number of publications, respectively. These results reflect the social, environmental, psychological, and physical dimensions of COVID-19-related health disparities. 

**Figure 1:**
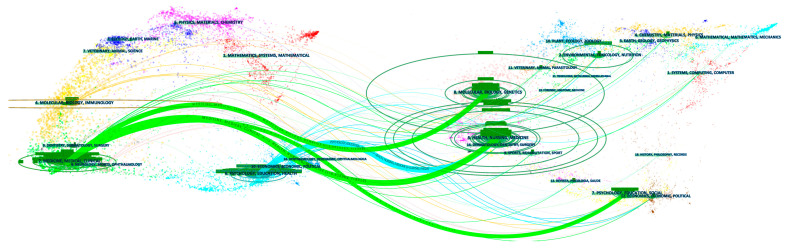
A subject matter overlay of publications on COVID-19-related health disparities. Note (s): wavelike curves represent citation links. They are colored by their source clusters. The dots in the clusters represent journals. The color of a dot denotes its Blondel cluster membership. The Blondel cluster membership is obtained by the Blondel algorithm [29].

To explore the citing trajectory of the 2282 publications at the discipline level, we used CiteSpace to construct a dual-map overlay of the journals (see Figure 1). Dual-map overlay is a method of portfolio analysis that depicts the sources (citing articles) and targets (cited references) of citations on a science map [28]. The dual-map base is generally used to make a single source overlay, organizational overlay, and subject matter overlay [28]. In the current study, the subject overlay showed from which discipline a citation is originated and which target discipline it pointed to. This allowed us to view the interdisciplinary connections of the research from a macro perspective.

In Figure 1, the clusters on the left contain the citing journals (the journals that published 2282 source articles), and the clusters on the right consist of cited journals (the journals of references cited by source articles). Each cluster refers to one discipline.

Publications that focused on COVID-19-related health disparities primarily originate from two disciplines. One is “medicine and medical and clinical” research; the other is “psychology, education, and health”. There were four trajectories from the citing journals to cited journals. Three of them were green and one was cyan. The “medicine and medical and clinical” research cluster of citing journals was mainly connected to the clusters of “molecular biology and genetics”; “health, nursing, and medicine”; and “psychology, education, and social science”. The cluster of “psychology, education, and health” was mainly connected to the cluster of health, nursing, and medicine. From the perspective of policy making or economics, there may be a gap between the discipline of medicine (“medicine and medical and clinical”), psychology (psychology, education, and health), and the discipline of economics (economics, economic and political) in studying the COVID-19-related health disparities. 

### 3.2. Analysis of the Distribution of Countries or Regions

According to WOS statistics, scholars from 116 countries or regions produced 2282 citing articles. Table 2 displays the 10 countries or regions with the highest number of publications, their share in the total number of publications, and citation frequencies. The country with the highest publication output was the United States, followed by England and Canada. All three countries or regions accounted for more than 100 articles each. The highest citation rate was also observed in the United States, which was followed by England and Australia. These three had the highest citation frequencies among all countries or regions. Meanwhile, authorities in some emerging-market countries, such as China, Brazil, and India, also focused on health disparities during the COVID-19 pandemic. As far as the number of published articles is concerned, those countries were ranked fifth, sixth, and tenth in the world, respectively.

**Figure 2:**
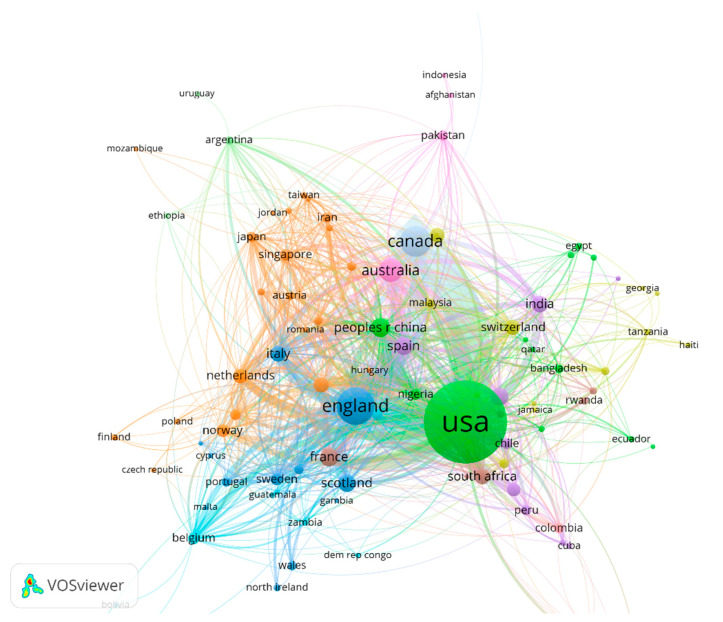
The co-operation networks of countries or regions. Note(s): Colors represent clusters, the thickness of linked lines represents intensity of co-operation, and node sizes represent the number of publications.

To make the figure more readable, the maximum number of countries per document was set to 25. The normalization (According to the VOSviewer manual, the normalization method is usually “used as input for the VOS layout technique and the VOS clustering technique”. There are three methods to normalize the strength of the links between items, including association strength, fractionalization, and linLog/modularity) method used in this paper was association strength to make the picture more readable. There were 99 countries or regions with strong associations, as evidenced in Figure 2. VOSviewer divided them into 12 clusters according to strength of association, with different colors representing different clusters (see Figure 2). In the cooperation network, the United States was in the core position, and the range of countries or regions that it cooperated with was the widest. This can be observed in Table 3. The USA had the most links and largest total link strength. Meanwhile, England and Canada, respectively, ranked the second and third highest in total link strength. However, the countries in Table 3 are different from the countries in Table 2. Although India and Scotland had the highest ranks in Table 2, they were not shown in Table 3 due to their low cooperation strength. 

The most intensive co-operations of the United States were with Canada and England. Among the emerging markets, China and India were also prominent in the collaboration networks. They not only produced a high volume of publications but they also collaborated extensively and intensively with most developed countries.

**Figure 3:**
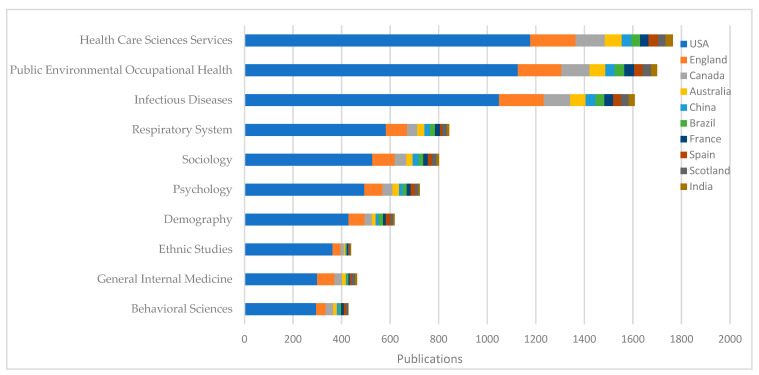
Country or region distribution of each research category.

At the same time, by using the top 10 research directions in Table 1 as a classification, we counted the number of publications in these areas for the 10 countries in Table 2, as shown in Figure 3. We found that these 10 countries generally focused on three areas of health disparities research during the COVID-19 pandemic, namely health care sciences and services, public environmental occupational health, and infectious diseases. Because the coronavirus is a type of respiratory disease, the disparity issues that it touches upon are closely related to biological factors in respiratory diseases. COVID-19 is also connected to health disparities that concern psychological, democratic, and other related social issues. In all these areas, the United States was the country with the highest number of publications. Other developed countries or regions accounted for an approximately equal share of the publications in each area. Emerging-market countries accounted for a significantly lower share of the publications in ethnic studies and general internal medicine than in other research areas. This finding may be due to cultural and political differences between countries or regions, which precipitate differences in research directions. 

### 3.3. Analysis of the Distribution of Journals

Table 4 displays the 10 most productive journals, in terms of the number of publications. These journals published a total of 341 papers, accounting for approximately 15% of the total number of citing articles (n = 2282). The top journal, in terms of the number of publications, was the *International Journal of Environmental Research and Public Health*, followed by *The Lancet*, the *Journal of Racial and Ethnic Health Disparities*, and others.

Table 5 presents the 10 most cited journals. The papers published in these journals had a strong impact in their field of research. Among them, *JAMA—Journal of the American Medical Association* and *The Lancet* both had more than 1000 citations and were among the top journals, in terms of the impact factor in the last five years.

**Figure 4:**
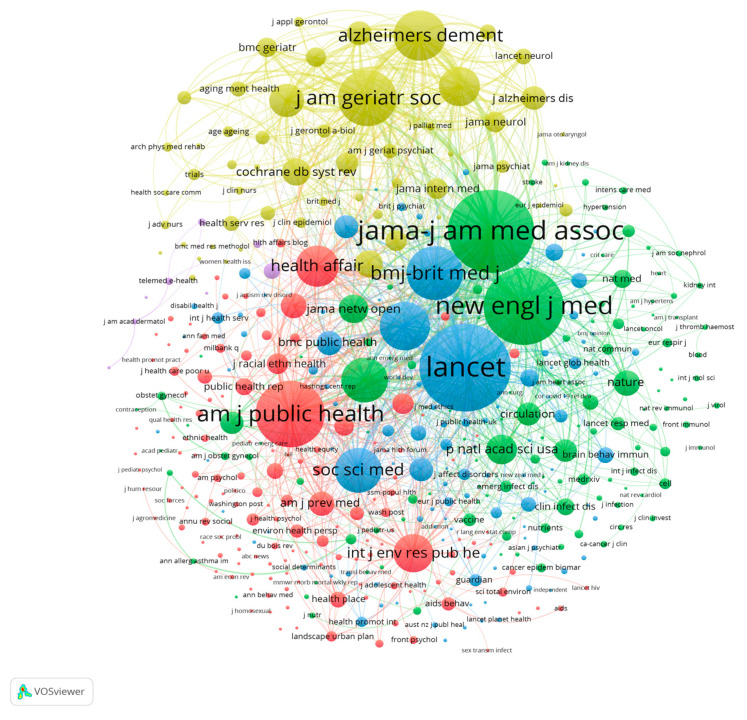
Co-citation network of journals (five clusters of 439 items). Note(s): Colors represent clusters, the thickness of linked lines represents the intensity of co-citation, and node sizes represent citation frequency.

We also analyzed the co-citations of journals. Cited references that were cited by 2282 source articles constituted a journal co-citation network. There were 24,516 source journals for the cited references. In order to obtain readable figure, we chose the journals with more than 20 citations. Then, there were 439 journals meeting the threshold. Finally, we constructed the co-citation networks of these journals and formed five clusters, as shown in Figure 4. Nodes with the same color represent the same cluster. In the green cluster, *JAMA—Journal of the American Medical Association* and the *New England Journal of Medicine* occupied the key positions. In the blue cluster, *The Lancet* and *BMJ—British Medical Journal—*were in relatively important positions. The *American Journal of Public Health*, the *International Journal of Environmental Research and Public Health*, and *Health Affairs* were central in the red cluster. In the yellow cluster, the *Journal of the American Geriatrics Society* and *Alzheimer’s & Dementia* were at the core. These findings indicate that the papers that were published in these journals made important contributions to the study of health disparities and COVID-19.

### 3.4. Analysis of Authors

**Figure 5:**
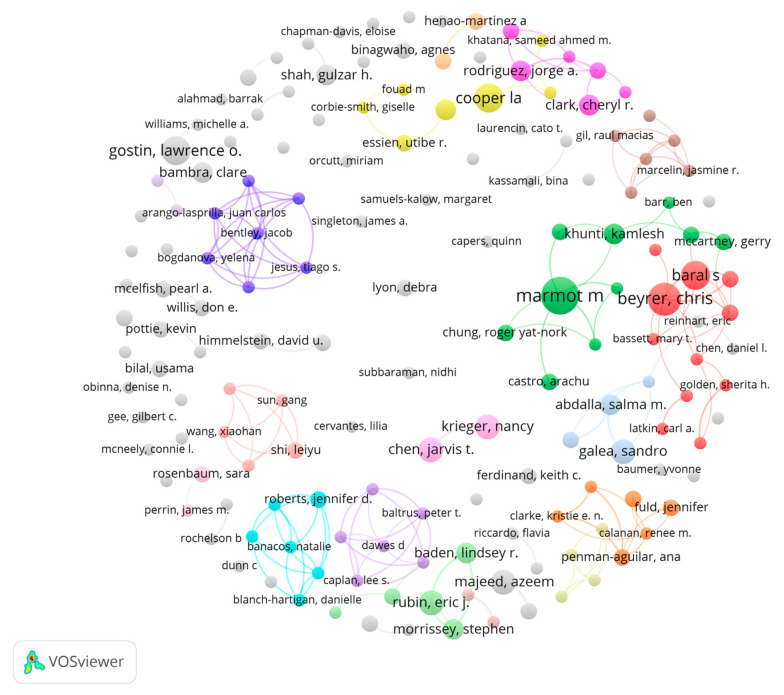
Co-authorship network. Note(s): Colors represent clusters, linked lines represent co-operative relationships, and node sizes represent publication numbers.

Table 6 displays the 10 authors with the highest number of publications. The most productive author was Marmot (n = 9), followed by Beyrer (n = 8). To understand the relationship between these authors and their roles further, we constructed a co-authorship network using VOSviewer (see Figure 5). The network consists of 162 authors with three or more publications. The network exhibits clear clusters of co-operation. For example, among the 10 most productive authors, Beyrer and Baral had a strong collaborative relationship. Chen and Krieger were two authors who collaborated closely. 

**Figure 6:**
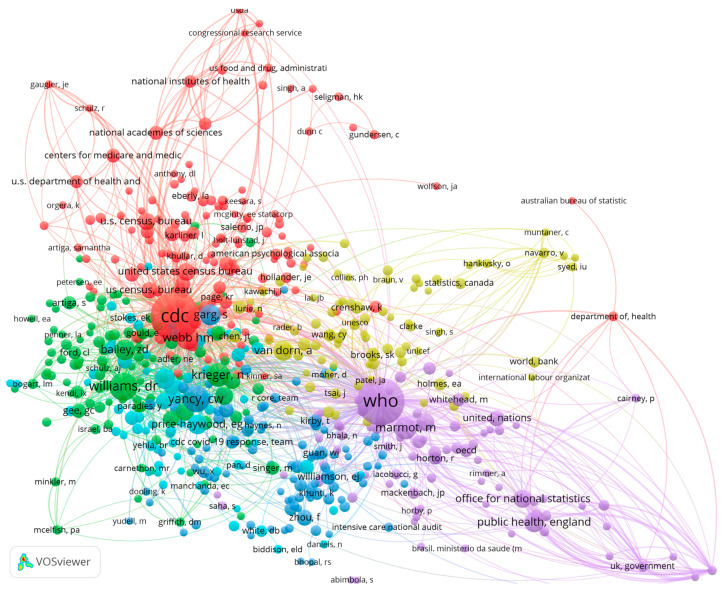
The authors co-citation network of cited references. Note(s): Color represents clusters, linked lines represent co-citation relationships, and node sizes represent citation frequencies.

The number of publications reflects an author’s workload and their interest in the field, but it does not capture the attention that their research has received or its contribution to other papers directly. For this reason, we performed an additional co-citation analysis of the references that were cited in articles. Core authors were mined further. Figure 6 presents the co-citation network that is based on 58,413 cited references. In total, 598 authors, including both institutional and individual ones, are shown in Figure 6. We used VOSviewer to classify these authors into six clusters based on association strength, with a minimum citation frequency of 10.

Figure 6 shows that the CDC and the World Health Organization (WHO) occupied the most central positions. Not only were they cited much more often than other authors, but they also had strong co-citation relationships. This finding indicates that the two organizations have provided very strong contributions to research on health disparities associated with the COVID-19 pandemic. Turning to individual authors, Yancy, Webb, Williams, Krieger, Marmot, and others occupied larger nodes than the rest of the individual authors, which suggests that the studies of these authors played a very important role in supporting existing research on health disparities and COVID-19.

### 3.5. Analysis of the Co-Occurrence of Keywords

**Figure 7:**
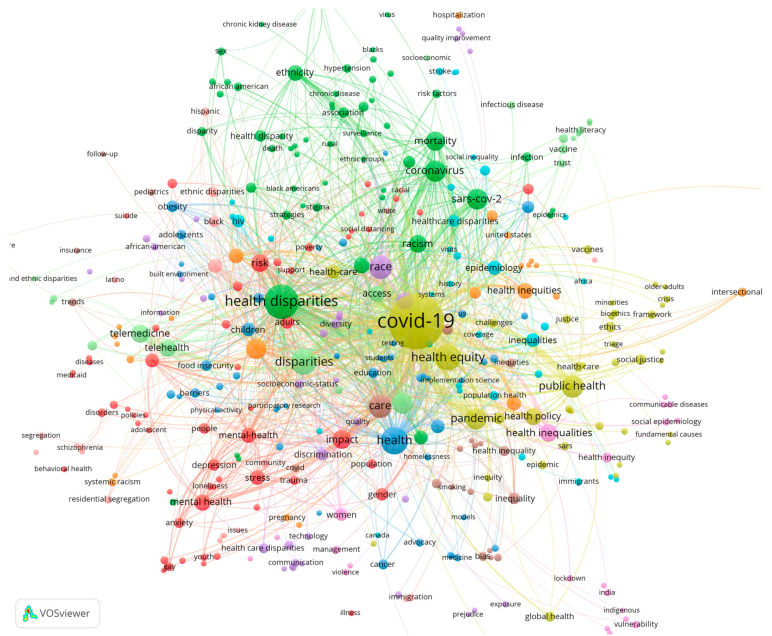
Keyword co-occurrence network. Note(s): The minimum number of occurrences of a keyword is 5. There are 384 keywords that meet the threshold.

The content of an academic article is distilled into its keywords. Therefore, analyzing co-occurrences of and connections between keywords can reveal trending topics, perspectives, and methods in research. We used VOSviewer to construct a keyword co-occurrence network for COVID-19-related health disparities, as shown in Figure 7. In that figure, keywords from similar categories are depicted in the same color, with 11 clusters in total. The larger a node, the more frequent the occurrence of the keyword that it represents. The lines that link the nodes indicate keyword co-occurrence relationships. “COVID-19” was the keyword with the highest number of occurrences. This is so because the present papers focused on health disparity studies that are related to the COVID-19 epidemic. We extracted the four most important categories of keywords from the 11 clusters, in line with Figure 7.

(1) “Health policy”, “public health”, “pandemic”, and such like. The COVID-19 outbreak exposed inequities in the public health system, which made it more difficult for governments to respond to public health emergencies. The studies in this category were dedicated to discussing how health authorities around the world can eliminate disparity and achieve equity at all stages of the fight against COVID-19 and thus propose effective public health policies [30]. For example, COVID-19 Medical Vulnerability Indicators have been proposed for use in public resource allocation in order to guarantee more equitable access to health care resources in infection-prone areas [31]. In addition, public regulatory policies may increase health disparities by themselves [32]. Public health policies should aim to provide support and assistance to vulnerable groups.
(2) “Racism”, “ethnicity”, ”race”, and such like. This part of the study focused on different ethnicities or populations as subjects of health disparities research. During the epidemic, COVID-19 outbreaks were concentrated among individuals in high-risk occupations, which usually involve vulnerable groups in society, further exacerbating socioeconomic and social inequalities in health [33]. Some scholars focused on the US health system. They argued that COVID-19 exposed and exacerbated pre-existing racial inequalities in American society and that it would have a very adverse impact on some minority communities, including black communities, Latino communities, immigrant communities, and Native American communities [34]. Health disparities between different populations have become the focus of public health policy in the context of the COVID-19 epidemic.
(3) “Mental health”, “stress”, “depression”, “gender”, “impact”, and such like. The relevant studies focused on the impact of epidemic prevention and control methods on human mental health, especially on differential impacts on the mental health of various groups, and revealed disparities in mental health. For example, social distancing during the COVID-19 outbreaks can exacerbate mental illness in the elderly and among vulnerable groups. Individuals with coronavirus pneumonia experienced severe psychological stress and faced a high risk of post-traumatic stress disorder. Pre-existing mental illnesses and disorders were aggravated by social isolation and lockdowns [35].
(4) “Disparities”, “telemedicine”, “telehealth”, “access”, and such like. The literature involving these keywords focused on disparities in access to health care, medications, etc., during the COVID-19 pandemic. Digital health emerged as an important factor and influenced health disparities during the COVID-19 outbreak [36]. The COVID-19 epidemic accelerated the development of digital health. Virtual features were useful in mitigating treatment interruptions and the reductions in access to medical care that resulted from the social distancing policies during the epidemic. As a result, some authors argued that digital technologies can improve access to care and governance in remote and medically backward areas, thereby improving equity [37]. However, poor health data have the potential to create a digital health divide instead, thus increasing health inequalities [38]. Therefore, eliminating health-data disparities and preventing the emergence of a digital health divide during the COVID-19 epidemic were important topics. These topics are also of considerable practical importance for the prevention and management of health disparities related to public health emergencies such as COVID-19.

**Figure 8:**
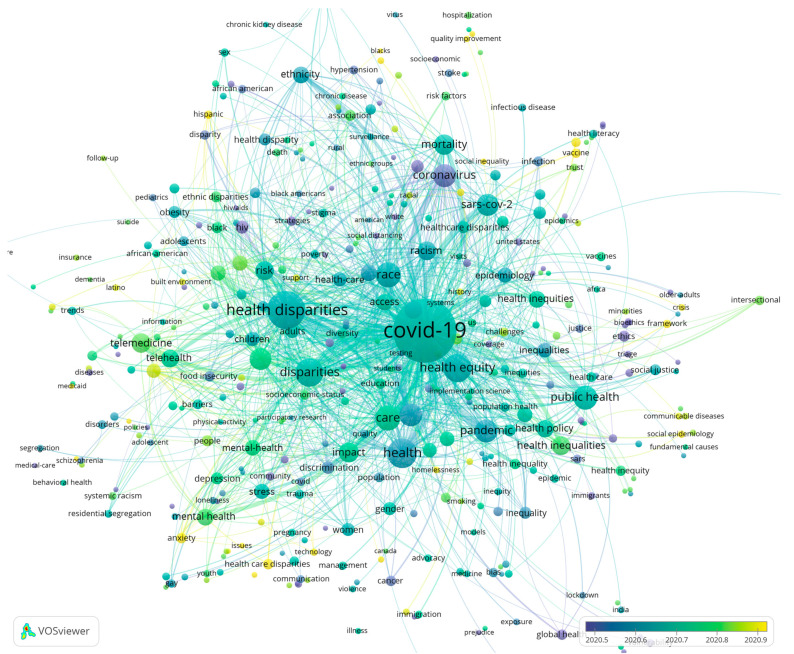
Overlay map of keyword co-occurrence network. Note(s): Color refers to the average publication year of keywords.

Figure 8 presents the average time trends in the keyword co-occurrence network. As can be seen from the figure, papers that referred to the two major categories mentioned above, (1) and (2), were published earlier on average. In other words, the outbreak of the COVID-19 epidemic caused scholars to become concerned about public policy, equity in resource allocation, and the racial health disparities that were influenced by the epidemic. The color of the two major categories of keywords (3) and (4) tends to turn yellow gradually, that is, average publication time is later for those categories. Progress in epidemic control and the promotion of digital technology saw scholars gradually begin to pay attention to mental health and digital health disparities. It is foreseeable that, in the future, these two topics will increasingly be subjected to extensive research. 

### 3.6. Analysis of Highly Cited Documents

We also analyzed articles with high citations. This section lists papers with more than 100 citations, of which there were 24 in total, as shown in Table 7. The high citation frequencies indicate that these studies are important and have received considerable attention in the academic community. 

The papers displayed in Table 7 can be divided into several main areas, the first being racial health disparities. Most of the 24 papers were on that subject. Some studies found evidence of potential health care racial disparities that affect African Americans, blacks, Latinos, etc., who were more likely to be infected with the coronavirus than whites and had higher mortality rates than those from predominantly white communities [39,40,42,50,58]. Differences in biomedical and social factors that are related to race contributed to differences in COVID-19 infection and mortality rates [44,45]. The social factors included living area, lifestyle, level of economic welfare, racism and discrimination, racial capitalism, health care access and governance, inequitable distributions of resources, the digital divide, food insecurity, housing insecurity, job risks, and more [40,48,49,51]. Some studies also constructed specific social vulnerability indices and identified health risk factors to analyze racial disparities during the pandemic [57]. In brief, COVID-19 has exposed and exacerbated the racial health inequities that those factors cause [39]. The pandemic has also increased the burden on society. For example, social distancing policies increased health inequalities for vulnerable groups and thus the burden that individuals and society must shoulder.

The second main area on which the papers focused was mental health disparities. Some studies suggested that the COVID-19 pandemic and resultant quarantine policies had the potential to exacerbate mental health disparities. Individuals at a high risk of mental problems were more likely to be negatively affected. Other studies have examined specific mental health problems among the public, among individuals with COVID-19, and among health care workers [35]. The results showed that public health policies should provide more mental health support to vulnerable groups.

The third focal area was gender health disparities. Studies found that men were twice as likely to be hospitalized with a confirmed COVID-19 infection than women, which supported other recent findings. For example, the CDC reported that although 49% of those diagnosed with COVID-19 were men, they accounted for 54% of hospitalizations according to case reports from China, Italy, and South Korea [47].

Fourth, some studies also reported health disparities between different occupations. For example, front-line professionals were more vulnerable to COVID-19 infection, such as those who work in retail, public transportation, and health care [34]. Moreover, most of those workers were from minority populations. These findings are closely related to racial health disparities. Finally, there were differences in the acceptance of the coronavirus vaccine. Studies have shown that vaccine acceptance varies with gender, age, race, and education, which indirectly contribute to health inequities [46].

### 3.7. Reference Co-Citation Analysis

Co-citation analysis is a method for identifying topics or knowledge bases in terms of a cluster of co-cited individual items. This method mainly includes two types: author co-citation analysis (ACA) and document co-citation analysis (DCA) [59]. DCA is a network of co-cited references that reveals more specific information than cited authors. We explored the author co-citation in Section 3.4. Therefore, in this part, we studied the knowledge base for COVID-19 related health disparities using the reference co-citation analysis. A total of 58,413 references from the 2282 citing articles were explored. These scholars and their research results have played important roles in promoting the development of the literature.

**Figure 9:**
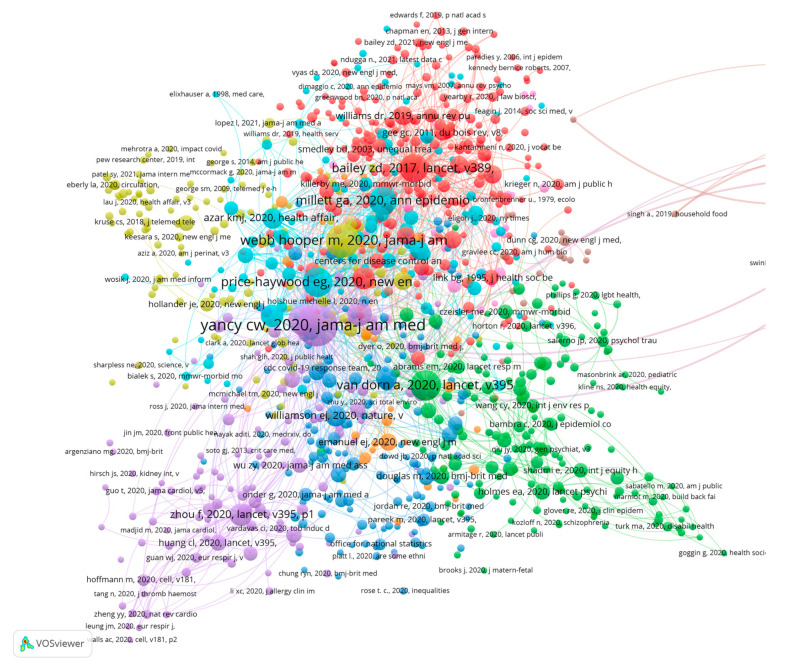
Co-citation network of cited references.

We selected references with more than five citations and obtained a total of 995 observations for the construction of the co-citation network. They were classified into nine clusters. Figure 9 reports the co-citation relationships and the number of citations for these references. The clusters in Figure 9 reveal the knowledge structures, and most of the references belong to five clusters—red, azure, yellow, purple, and green. Table 8 reports the top five highly co-cited references in each of the five clusters.

In the red cluster, the references studied the relationship between racism and health inequalities, and the interventions in racism-related health inequalities [45,60,61,62,63]. Table 8 reports the top five highly cited references in the red cluster. Bailey et al. (2017) focused on structural racism and health inequities in the United States [60]. Structural racism was defined as “the totality of ways in which societies foster racial discrimination through mutually reinforcing systems of housing, education, employment, earnings, benefits, credit, media, health care, and criminal justice”. (p. 1) They argued that structural racism would harm health disparities. As such, they proposed interventions to address structural racism. These interventions included the “place-based, multisector, equity-oriented initiatives”, “advocating for policy reform”, and “training the next generation of health professionals” [60]. COVID-19 exacerbated the social risks posed by structural racism. In response, Egede et al. (2020) proposed a six-pronged approach for addressing structural racism health disparities [62]: (1) change policies related to generating or maintaining structural racism; (2) establish cross-sectoral infrastructure and finance sharing mechanisms while integrating health interventions into cross-sectoral collaborative systems; (3) increase economic empowerment of vulnerable populations; (4) include community programs for establishing stable and supportive structures as part of pandemic recovery efforts; (5) implement health systems that build trust in vulnerable communities; (6) and establish interventions that target social risk factors.

In the green cluster, the references discussed the relationship between COVID-19 and inequality. On the one hand, COVID-19 and the responses to it will exacerbate health inequalities [8]. Van Dorn (2020) argued that the pandemic has worsened health inequalities in the United States, particularly racial health disparities, disparities between the health of the insured and the uninsured in rural areas, and public health disparities [34]. COVID-19 disproportionately affects vulnerable populations, including people with mental illness, people with physical disabilities, and those in digital poverty [64]. Furthermore, Douglas et al. (2020) argued that the responses to COVID-19 also widened health disparities through several mechanisms [32], including “economic effects, social isolation, family relationships, health related behaviors, disruption to essential services, disrupted education, transport and green space, social disorder, and psychosocial effects” [32]. On the other hand, pre-existing inequalities also exacerbated the spread of COVID-19. For example, poor populations are less informed, more densely populated, less resourced, and more difficult in terms of implementing social distance policies. Accordingly, poorer areas are more conducive to disease transmission [65]. 

In the yellow cluster, references mainly concerned the real-time data and interventions of health disparities among vulnerable populations during COVID-19. The classification of disadvantaged groups was mainly based on race, income, education, and household crowding [40,64,66]. The most cited article was written by Webb (2020). This article offered a viewpoint that focused on the factors influencing racial health disparities. The study noted the need for public health agencies to collect data by race and thus suggested guidance for regulatory policy and prevention interventions for COVID-19 [40]. In this cluster, some scholars studied telemedicine-related health disparities among disadvantaged populations. Nouri et al. (2020) discussed addressing equity in chronic disease management in telemedicine during the pandemic [67]. They argued that telemedicine may increase inequities in COVID-19-related care for vulnerable populations with limited digital literacy and access capabilities because disadvantaged groups are more likely to face digital barriers. As such, the article proposed four key actions to reduce telemedicine-induced health disparities: first, identify disparities in access to telemedicine; second, mitigate digital literacy and resource barriers by education and training of digital skills; third, remove health system-created barriers by offering various visit methods; and fourth, provide inclusive telemedicine.

In the purple cluster, co-cited references focused on monitoring or discussing the risk factors of health disparities in patients with COVID-19. For example, these studies consistently monitor and report hospitalization rates, clinical characteristics, and outcomes of hospitalized patients with different ages, genders, races, concomitant comorbidities, and socioeconomic status [39,42,66,68,69]. The analysis of the factors influencing health disparities under COVID-19 is important for scientific planning and for guiding the effective allocation of health care system resources. Within this cluster, Yancy (2020), published in *JAMA-Journal of the American Medical Association*, had the highest citation frequency. The article noted that the scourge of COVID-19 has further exacerbated health care disparities [39]. In the United States, African Americans or blacks are more likely to be infected with COVID-19 and more likely to die. This is partly caused by concomitant comorbidities. On the other hand, it is also due to socioeconomic factors. For example, most blacks live in areas of poverty, with high housing density, high crime rates, and poor access to healthy food. Moreover, policies such as social distancing have an even greater negative impact on the livelihoods of poor people, as they are less able to do their jobs by working from home or telecommuting. As such, Yancy reflected deeply on the risk factors for health disparities during COVID-19 and emphasized that these factors have also persisted throughout history. He suggested the urgent need for public health system changes to address health care disparities [39]. This clustering is similar to the yellow clustering. However, this cluster focused more on the factors influencing health disparities, while the yellow cluster focused more on interventions for health disparities.

The blue cluster is similar to the yellow cluster. However, its primary topic concerns the health disparities among different races, such as black and white [9,70], ethnic minority groups, and other races [44,47]. Millett (2020) used data on COVID-19 cases and deaths to conclude that black communities have faced a higher probability of being at risk of infection or death during the COVID-19 pandemic [70]. Price-Haywood (2020) examined hospitalization disparities and mortality differences between black and white patients with COVID-19 [9].

## 4. Discussion and Conclusions

### 4.1. Discussion

During the pandemic, the health disparities related to COVID-19 have been exacerbated and caused wide concern. More than two thousand articles were published in less than 22 months (January 2020‒October 2021). Based on the analysis of research categories, these studies had significant interdisciplinary characteristics. For example, epidemiology, psychology, biomedicine, sociology, and other disciplines. However, the analysis of the dual-map overlay of the journals suggested that fewer links may exist between the available studies and disciplines such as economics. The study of COVID-19-related health disparities from an economic perspective deserves further depth.

The USA was the most productive country, especially the CDC, an institution from the U.S. Together with the WHO, the CDC occupied an absolute core position in the authors co-citation network. Moreover, the U.S. has the broadest scope and intensity of cooperation. Scholars from the United States have strong collaborative relationships with most countries in the world. This may be mainly due to the strong scientific resources and the large number of scientific talents in the United States [74]. 

Another reason may be that the United States, as a developed market economy with a large gap between rich and poor, has its own more pronounced health disparity problem. The outbreak of COVID-19 has exacerbated the existing health disparity problem. Therefore, scholars from the U.S. believe that COVID-19 provided a window to call for much-needed reform of the system to eliminate health disparities. 

Among the emerging markets, China was also prominent in the collaboration networks. It not only produced a high volume of publications but also collaborated extensively and intensively with most developed countries. In addition, compared to developed countries, emerging market countries have paid less attention to racial health inequities. This may be because, at the current stage of development, they are more concerned with health disparities caused by socioeconomic and other factors. 

The *JAMA—Journal of the American Medical Association* (60.15) and *The Lancet* (77.24) had more than 1000 citations and rank among the top in average citations. Among the highly cited articles, those with a citation frequency of more than 500 were from these two journals. This indicates that the papers published in these two journals have made important contributions in this research area.

This paper not only shows how scholars and their networks publish their works by analyzing the distribution of publications, but also details several of the most popular COVID-19-related health disparity research topics and directions through keyword co-occurrence, highly cited articles, and reference co-citation analysis. According to the keyword co-occurrence, the most popular themes were racial, mental, and digital health disparities and the intervention policies of health disparities. Among them, the mental and digital health disparities were emerging topics. The research directions of all citing articles and cited references could be grouped into three categories, which can also serve as fields for continued in-depth research in the future.

(1) The main manifestations of health disparities under the influence of COVID-19. Studies have been conducted to report health disparities based on real-time data focusing on groups differing by ethnicity, mental status, occupation, gender, education, income, concomitant comorbidities, and others [34,35,39,40,42,66,68,69]. The results showed that COVID-19 has caused a disproportionate increase in infection rates in vulnerable populations. These studies have particularly focused on racial health disparities during the pandemic. We found that authors from developed countries were more concerned about racial health disparities than authors from emerging-market countries.
(2) Factors influencing health disparities during the COVID-19 pandemic. Based on the analysis in Section 3, the following factors have received significant attention and discussion: public health policy of COVID-19 [8,32], racism [60], digital divide [67], socioeconomic status [39]. Specifically, the responses to COVID-19 may exacerbate health disparities through social isolation, economic effects, etc. Structural racism and digital divide would also affect the access to health care for disadvantage groups, and socioeconomic status affects the ability and resources of different populations to resist COVID-19.
(3) Interventions targeting health disparities during COVID-19 pandemic. The analysis of the features and factors of health disparities during the pandemic is important for the effective implementation of health care regulatory policy and allocation of public health resources. For example, COVID-19 medical vulnerability indicators have been proposed for use in public resource allocation to guarantee more equitable access to health care resources in infection-prone areas [31]. Egede et al. (2020) proposed a six-pronged approach to addressing structural racism that exacerbates health disparities [62]. It is necessary for public health regulatory agencies to collect data by race and thus establish guidelines for policy and prevention interventions targeting COVID-19 [40]. Some scholars suggested four key actions to reduce telemedicine-induced health disparities [67].

Based on research directions, COVID-19 exacerbates multiple inequalities, which has some policy implications. In the event of an unexpected public health emergency, such as the COVID-19 pandemic, regulatory agencies can help vulnerable populations protect themselves by implementing effective intervention programs and resource allocation, thereby preventing them from suffering more severe losses than other groups.

### 4.2. Conclusions

In this paper, we used VOSviewer and CiteSpace to analyze the distribution of publications, research trends, and prominent topics in research on the health disparities influenced by COVID-19. We advanced three main conclusions. 

First, we summarized the main research directions in the field of health disparities related to the COVID-19 pandemic. The research category that attracted the most publications is health care sciences and services. The literature in this area is mainly based on the disciplines of molecular biology, health nursing, and psychology. 

Second, we identified the countries, journals, and authors (both individual and institutional) that have made major contributions to the field. The country with the most articles was the United States. The journal with the most publications was the *International Journal of Environmental Research and Public Health*, and *JAMA—Journal of the American Medical Association* had the highest number of average citations. The most influential institutional authors were the CDC and the WHO, and the most influential individual authors were Yancy, Webb, Williams, Krieger, and Marmot.

Third, we identified the trends and directions of health disparity related to the impact of the COVID-19 pandemic by keyword co-occurrence analysis, highly citations articles analysis, and reference co-citation analysis. The trending topics mainly concern racial, mental, and digital health disparities. The research directions have focused on three areas: the features of health disparities during COVID-19, factors influencing health disparities during COVID-19, interventions for health disparities during COVID-19. 

This paper also has some limitations. Due to the length of the paper, the data were drawn mainly from English-language papers in the WOS database, and other articles from other databases were not included. In the future, other samples can be selected to conduct comparative studies. Furthermore, we focused on health disparities during the COVID-19 pandemic, and we did not analyze the literature on the way public health regulatory policies eliminate health disparities. Research on the public health regulatory policies that were enacted during the COVID-19 pandemic, especially on those regulatory policies that seek to eliminate health disparities, will be conducted in the future.

## Figures and Tables

**Table 1:** Top 10 research categories for health disparity and COVID-19.

Research Categories	Publications	Percentage of Publications (%)
Health care sciences and services	1620	70.990
Public environmental occupational health	1573	68.931
Infectious diseases	1481	64.899
Respiratory system	777	34.049
Sociology	722	31.639
Psychology	666	29.185
Demography	580	25.416
General internal medicine	451	19.763
Ethnic studies	400	17.528
Behavioral sciences	396	17.353

Note(s): The data were retrieved from the WOS. All tables in the text have the same data source unless stated otherwise. The total number of publications (*n* = 2282) was used to calculate the percentage of publications.

**Table 2:** Top 10 productive countries.

Country/Region	Publications, *n* (%)	Citations, *n* (%)
USA	1620 (70.99)	14,224 (79.04)
England	261 (11.437)	2843 (15.80)
Canada	170 (7.45)	1212 (6.74)
Australia	92 (4.032)	1571 (8.73)
China	54 (2.366)	1050 (5.83)
Brazil	51 (2.235)	477 (2.65)
France	51 (2.235)	1099 (6.11)
Spain	48 (2.103)	565 (3.14)
Scotland	44 (1.928)	1203 (6.69)
India	41 (1.797)	627 (3.48)

Note(s): The total number of publications (n = 2282) was used to calculate the countries’ shares. The total frequency of citations (n = 17,995) was used to calculate the percentage of citations.

**Table 3:** The countries cooperation network ranked by total link strength.

Country	Links	Total Link Strength
USA	79	625
England	72	435
Canada	61	268
Australia	55	245
Italy	46	152
China	49	150
Spain	50	146
Netherlands	40	126
Brazil	48	116
France	36	108

Note(s): the links refer to the volume of countries that one country cooperates with.

**Table 4:** Top 10 most productive journals for research on health disparity and COVID-19 (*n* = 2282).

Journals	Publications, *n* (%)	IF-5 Years	Citations
International Journal of Environmental Research and Public Health	49 (2.15)	3.79	317
The Lancet	43 (1.88)	77.24	1379
Journal of Racial and Ethnic Health Disparities	39 (1.71)	2.32	509
BMJ—British Medical Journal	36 (1.58)	15.88	494
American Journal of Public Health	33 (1.45)	8.41	134
JAMA—Journal of the American Medical Association	32 (1.40)	60.15	2199
Frontiers in Public Health	30 (1.32)	4.02	92
International Journal for Equity in Health	27 (1.18)	3.81	213
JAMA Network Open	26 (1.14)	8.49	167
Journal of General Internal Medicine	26 (1.14)	6.07	210

Note(s): IF-5 years refer to impact factor in the last five years.

**Table 5:** Top 10 journals with high citation frequencies.

Journals	Publications	Citations	Avg. Citation	IF-5 Years
JAMA—Journal of the American Medical Association	32	2199	68.72	60.15
The Lancet	43	1379	32.07	77.24
New England Journal of Medicine	21	620	29.52	89.68
Journal of Racial and Ethnic Health Disparities	39	509	13.05	2.32
BMJ—British Medical Journal	36	494	13.72	13.51
MMWR—Morbidity and Mortality Weekly Report	25	474	18.96	12.99
Lancet Psychiatry	7	404	57.71	26.93
Health Affairs	21	331	15.76	7.03
International Journal of Environmental Research and Public Health	49	317	6.47	3.789
Clinical Infectious Diseases	8	308	38.5	9.60

Note(s): Avg. is the abbreviation for average.

**Table 6:** The 10 most productive authors.

Author	Documents	Citations	Total Link Strength
Marmot, M.	9	54	11
Beyrer, C.	8	120	11
Baral, S.	7	158	7
Cooper, L. A.	7	188	2
Gostin, L. O.	7	20	2
Chen, J. T.	6	133	3
Galea, S.	6	81	11
Krieger, N.	6	166	3
Majeed, A.	6	87	4
Rubin, E. J.	6	6	11

Note(s): Total link strength was calculated by VOSviewer on the basis of co-authorship networks.

**Table 7:** The list of articles with citation frequencies larger than 100 (c = 17995).

Title	Authors	Citations, c (%)
COVID-19 and African Americans	Yancy, C. W. [39]	817 (4.54)
COVID-19 and racial/ethnic disparities	Webb Hooper, M., et al. [40]	648 (3.60)
Dementia prevention, intervention, and care: 2020 report of the Lancet Commission	Livingston, G., et al. [41]	502 (2.79)
COVID-19 exacerbating inequalities in the US	van Dorn, A., et al. [34]	451 (2.51)
How mental health care should change as a consequence of the COVID-19 pandemic	Moreno, C., et al. [35]	282 (1.57)
The COVID-19 pandemic: a call to action to identify and address racial and ethnic disparities	Laurencin, Cato T., et al. [42]	277 (1.54)
Variation in COVID-19 hospitalizations and deaths across New York city boroughs	Wadhera, R. K., et al. [43]	267 (1.48)
The Disproportionate impact of COVID-19 on racial and ethnic minorities in the United States	Tai, Don Bambino Geno, et al. [44]	259 (1.44)
Mitigating the wider health effects of COVID-19 pandemic response	Douglas, M., et al. [32]	256 (1.42)
Racial health disparities and COVID-19-caution and context	Chowkwanyun, M., et al. [45]	226 (1.26)
Determinants of COVID-19 vaccine acceptance in the US	Malik, Amyn, A., et al. [46]	211 (1.17)
Disparities in outcomes among COVID-19 patients in a large health care system in california	Azar, Kristen, M. J., et al. [47]	180 (1.00)
Disparities in the population at risk of severe illness from COVID-19 by race/ethnicity and income	Raifman, Matthew, A., et al. [48]	155 (0.86)
The COVID-19 pandemic and health inequalities	Bambra, C., et al. [8]	142 (0.79)
Ethnic and regional variations in hospital mortality from COVID-19 in Brazil: a cross-sectional observational study	Baqui, P., et al. [49]	137 (0.76)
Disparities in incidence of COVID-19 among underrepresented racial/ethnic groups in counties identified as hotspots during june 5-18, 2020-22 states, february-june 2020	Moore, Jazmyn, T., et al. [50]	133 (0.74)
Racial capitalism: a fundamental cause of novel coronavirus (COVID-19) pandemic inequities in the United States	Laster, P., et al. [51]	125 (0.69)
Combating COVID-19: health equity matters	Wang, Z., et al. [52]	122 (0.68)
COVID-19-implications for the health care system	Blumenthal, D., et al. [53]	116 (0.64)
The neglected health of international migrant workers in the COVID-19 epidemic	Liem, A., et al. [54]	115 (0.64)
Health equity and COVID-19: global perspectives	Shadmi, E., et al. [55]	114 (0.63)
Characterizing the Impact of COVID-19 on men who have sex with men across the United States in April, 2020	Sanchez, T. H., et al. [56]	109 (0.61)
Social vulnerability and racial inequality in COVID-19 deaths in Chicago	Kim, S. J., et al. [57]	107 (0.59)
Ethnic and socioeconomic differences in SARS-cov-2 infection: prospective cohort study using UK Biobank	Niedzwiedz, C. L., et al. [58]	102 (0.57)

**Table 8:** The top 5 highly co-cited references in five largest clusters.

Cluster	Cited References	Total Link Strength	Citations
Red	Structural racism and health inequities in the USA: evidence and interventions [60]	987	90
	Racial Health Disparities and COVID-19—Caution and Context [45]	575	57
	Racism and Health: Evidence and Needed Research [61]	514	41
	Structural Racism, Social Risk Factors, and COVID-19—A Dangerous Convergence for Black Americans [62]	548	39
	COVID-19 and Health Equity—A New Kind of “Herd Immunity” [63]	310	36
Green	COVID-19 exacerbating inequalities in the US [34]	1048	106
	Multidisciplinary research priorities for the COVID-19 pandemic: a call for action for mental health science [71]	234	30
	Mitigating the wider health effects of COVID-19 pandemic response [32]	233	29
	The COVID-19 pandemic and health inequalities [8]	147	27
	Why inequality could spread COVID-19 [65]	258	25
Yellow	COVID-19 and Racial/Ethnic Disparities [40]	1060	127
	Addressing Equity in Telemedicine for Chronic Disease Management During the COVID-19 Pandemic [67]	119	27
	Virtually Perfect? Telemedicine for COVID-19 [72]	93	22
	Racial, Economic, and Health Inequality and COVID-19 Infection in the United States [64]	238	20
	Revealing the Unequal Burden of COVID-19 by Income, Race/Ethnicity, and Household Crowding: US County Versus Zip Code Analyses [73]	205	17
Purple	COVID-19 and African Americans [39]	1700	197
	Hospitalization Rates and Characteristics of Patients Hospitalized with Laboratory-Confirmed Coronavirus Disease 2019—COVID-NET [68]	867	107
	The COVID-19 Pandemic: A Call to Action to Identify and Address Racial and Ethnic Disparities [42]	699	67
	Clinical course and risk factors for mortality of adult inpatients with COVID-19 in Wuhan, China: a retrospective cohort study [66]	580	58
	Clinical features of patients infected with 2019 novel coronavirus in Wuhan, China [69]	366	30
Blue	Hospitalization and Mortality among Black Patients and White Patients with COVID-19 [9]	1048	100
	Assessing differential impacts of COVID-19 on black communities [70]	929	89
	Variation in COVID-19 Hospitalizations and Deaths Across New York City Boroughs [43]	530	57
	Disparities in Outcomes Among COVID-19 Patients in a Large Health Care System In California [47]	545	48
	The Disproportionate Impact of COVID-19 on Racial and Ethnic Minorities in the United States [44]	333	40

## Data Availability

No data statement.
